# The efficacy and safety of atogepant for the prophylactic treatment of migraine: evidence from randomized controlled trials

**DOI:** 10.1186/s10194-022-01391-2

**Published:** 2022-01-29

**Authors:** Xinyu Tao, Zeya Yan, Jiahao Meng, Wei Wang, Qiling Dai, Qiufeng Zhou, Zhifeng Wang, Zhong Wang

**Affiliations:** 1 Department of Neurosurgery & Brain and Nerve Research Laboratory, The First Affiliated Hospital of Soochow University, Suzhou, 215006 Jiangsu Province China; 2 Department of Neurosurgery, Suzhou Ninth People’s Hospital, Suzhou, China; 3 Department of Neurosurgery, The First People’s Hospital of Nantong City, Nantong, Jiangsu Province China

## Abstract

**Background:**

Migraine is a common neurovascular disorder that has a severe impact on the individual daily life. Atogepant (AGN-241689) is an orally ingested, small-molecule drugs belonging to calcitonin gene-related peptide receptor antagonist, which has been initiated for the prophylactic treatment of migraine. However, there is no comprehensive literature to study the efficacy and safety of atogepant for the treatment of migraine. In this article, we present a meta-analysis of the available studies.

**Methods:**

MEDLINE, Embase, Cochrane Library and ClinicalTrials.gov were searched before October 20, 2021 for any relevant literature. Eventually, three randomized clinical trials (RCTs) with 2,466 patients were included in our study.

**Results:**

We pooled 2,466 patients from 3 RCTs and primary outcome was mean monthly migraine days, the secondary endpoints were monthly headache days, acute medication use days per month and ≥ 50% reduction in monthly migraine days, baseline to end of trials. It was found that atogepant (10 mg, 30 mg, 60 mg once a day) led to a significant reduction in monthly migraine days (*P* < 0.00001, *P* < 0.00001, *P* = 0.007), monthly headache days (*P* < 0.00001, *P* < 0.00001, *P* = 0.001), and monthly medication use days (*P* < 0.00001, *P* < 0.00001, *P* = 0.0001), and an increase in the proportion of people with ≥ 50% reduction in monthly migraine days (*P* = 0.0008, *P* = 0.02, *P* = 0.04) in comparison with placebo. Moreover, there were no significant differences (*P* > 0.05) in outcomes of adverse events between atogepant and placebo.

**Conclusions:**

Atogepant has shown good efficacy and safety in the prophylactic treatment of migraine, and further studies are expected.

**Supplementary Information:**

The online version contains supplementary material available at 10.1186/s10194-022-01391-2.

## Introductions

Migraine is a frequently occurring neurovascular disorder featured of unilateral and repetitious attacks of pulsating headaches, which are exacerbated by daily activities and may accompanied by systemic symptoms such as nausea, vomiting, photophobia and phonophobia [1], that could affect the patients' ability to perform activities of daily living. Young and middle-aged people are the main population of migraine attack, particularly with a higher prevalence in women [2]. At least 1 billion people worldwide suffered from migraine in their lifetime [3]. Globally, Migraine imposes a significant burden on individuals and society due to the physiological pain, mobility discomfort and the cumulative cost of treatment [2, 4, 5].

Many classifications have been applied to the division of migraine subtypes. Among these, it is of great significance for the health management strategies of patients to distinguish between the episodic and chronic forms of this disease. According to The International Classification of Headache Disorders, 3rd edition, episodic migraine (EM) is referred to the headache occurring on fewer than 15 days per month, lasting 4–72 h every time. Chronic migraine (CM), is defined as a headache that occurs at least 15 days per month, lasts for more than 3 months and has to present migraine characteristics on at least 8 days per month [6, 7].

When it comes to the management of migraine, two important aspects are acute treatment and preventive treatment. Acute treatment is essential, which gets interventions during a migraine acute attack to obtain relief. While preventive treatment depends on the frequency and severity of the migraine attack, taking into account the effects of adverse reactions [8]. CM, which may derive from episodic migraine, has a higher rate of disability and worse result of therapy, and is more associated with neurological dysfunction [7, 9]. According to previous epidemiological studies, about 38% migraineurs need to be preventively treated, with a view to reduce the frequency of migraine attacks and delay disease progression [10, 11].

A variety of medications have been used for the preventive treatment of migraine, but these treatments are not effective enough or not tolerated by some patients [12]. The pharmacological effects of drugs cannot be separated from the study on the pathophysiology of migraine. It is commonly accepted that the pathogenesis of migraine relates to abnormal activation and sensitization of the vascular pathways of the trigeminal nervous system [13]. In recent years, with the discovery that migraine attacks may be related to the provocation action of calcitonin gene-related peptide (CGRP) [14], the drugs relating CGRP ligand and receptor have become a new hotspot for clinical use, especially in the acute treatment of migraine attack, and some of them also have been approved for prophylactic treatment one after another, including several CGRP function-blocking monoclonal antibodies (MAbs), erenumab (the recommended dose is 70 mg or 140 mg, two consecutive injections of 70 mg, by subcutaneous injection once a month), fremanezumab (subcutaneous injection once a month or once every 3 months), galcanezumab (the initial loading dose is 240 mg, two consecutive 120 mg, followed by 120 mg per month by subcutaneous injection) and eptinezumab (recommended to be administered 100 mg by intravenous injection every 3 months), etc. [15–20]. Although these MAbs have already been available for the prophylactic treatment of migraine, their subcutaneous or intravenous mode of administration caused a degree of inconvenience to patients.

Other treatments such as antidepressants, anticonvulsants, antihypertensive drugs and onabotulinumtoxinA were also recommended for clinical application, but all drugs mentioned above were originally developed as non-specific therapies [1, 21]. When it comes to this issue, we must refer to the other drugs acting on the CGRP pathway, namely CGRP receptor antagonists (gepants). Unlike the preventive MAbs, gepants are mainly taken in forms of pill, nasal spray, orally disintegrating tablet (ODT) [22]. There are currently 2 gepants including ubrogepant and rimegepant (ODT) approved by the FDA respectively in 2019 and 2020 for acute migraine treatment [22]. Subsequently, rimegepant in tablet form was approved for migraine prevention on May 27, 2021, the only gepant that could be used for both acute and preventive migraine treatment. Atogepant became the second FDA-approved oral gepants for migraine prevention which gained approval on 28 September 2021 in the USA, which is also the first oral drug to be exclusively developed for the preventive treatment of episodic migraine [23]. According to the official instructions, the recommended dose is 10,30 or 60 mg once a day [23]. However, a systematic evaluation for the efficacy and safety of atogepant has not yet been performed. In this regard, we conducted a study to discuss the preventive effects of atogepant at different doses through a meta-analysis.

## Methods

A meta-analysis was conducted in conformity with the Preferred Reporting Items for Systematic Reviews and Meta-Analyses (PRISMA) guidelines. Our study has not been registered.

### Search strategy

We systematically searched MEDLINE, Embase, Cochrane Library database and ClinicalTrials.gov for any relevant clinical trials published before October 20, 2021. Search terms included the following: migraine AND (atogepant OR AGN-241689). The reference lists and discussion sections of the identified studies and meta-analyses were searched for additional studies. After removing duplicate and irrelevant studies, two investigators manually screened each possible article by reading title, abstract, etc., to determine whether the study met the predefined inclusion criteria. Any divarication came to an agreement through enough discussion.

### Study selection

Studies were incorporated into our meta if (a) the type of studyies is RCT; (b) participants are adults, aged 18 to 80 years and diagnosed with migraine for at least one year; (c) patients had 4 to 14 migraine days monthly before the trials; (d) study used atogepant or placebo as intervention. Studies were excluded if (a) types of study were retrospective studies, cohort studies, and case reports; (b) active control was adopted (namely a known, effective treatment instead of compared with an experimental treatment).

### Data extraction

All the data were extracted independently by 2 investigators (XYT and ZYY) and any disagreements were settled through discussion. After carefully assessing and selecting, the basic information of the included trials (first author, year, number of NCT, countries, centers, publication, type of migraine and treatment group), patient characteristics (age range, mean age and gender), study period and outcome events were used to extract the data (Table 1).

**Table 1:** Characteristics of the included studies and outcome events

Study	Countries	Centers	Publication	Type of migraine	Treatment group, (No. of participants)	Total number	Age range	Female (%)	Mean age ± SD (year)	Study period	Outcome Events
Goadsby et al 2020 (NCT02848326)	USA	78	Lancet Neurol	episodic	Atogepant 10 mg QD (93) vs.30 mg QD (183) vs.60 mg QD (186) vs.30 mg BID (86) vs.60 mg BID (91) vs. PLA (186)	825	18y-75y	10 mg QD: 88 30 mg QD: 91 60 mg QD: 84 30 mg BID: 85 60 mg BID: 91 PLA: 83	10 mg QD:39.4 ± 12.4 30 mg QD:41.0 ± 13.6 60 mg QD:40.4 ± 11.7 30 mg BID:38.5 ± 11.2 60 mg BID:39.7 ± 11.9 PLA:40.5 ± 11.7	12w	a b c d e
Ailani et al 2021 (NCT03777059)	USA	138	NEJM	episodic	Atogepant 10 mg QD (221) vs.30 mg QD (228) vs.60 mg QD (231) vs. PLA (222)	902	18y-80y	10 mg QD: 90.5 30 mg QD: 89.5 60 mg QD: 86.1 PLA: 89.2	10 mg QD:41.1 ± 12.0 30 mg QD:42.1 ± 11.7 60 mg QD:42.5 ± 12.4 PLA: 40.3 ± 12.8	12w	a b c d e f g h
Allergan et al 2021 (NCT03700320)	USA	112	/	episodic	Atogepant 60 mg QD	739	18y-80y	60 mg QD: 88.2 PLA: 87.8	60 mg QD: 41.1 ± 12.1 PLA: 42.5 ± 12.03	52w	a

*PLA* placebo, *w* weeks a Adverse Events (*AEs*), b monthly migraine days (*MMDs*), c Monthly headache days, d Monthly medication use days, e ≥ 50% reduction in monthly migraine days, f Score on Role Function–Restrictive domain of MSQ, g Score on Performance of Daily Activities domain of AIM-D, h Score on Physical Impairment domain of AIM-D

### Outcome measures

The primary efficacy outcome is mean monthly migraine days (MMDs), baseline to the end of trials. Secondary efficacy endpoints included: mean monthly headache days, acute medication use days per month and patients with a 50% reduction in migraine days from baseline (50% responder rate). Moreover, the adverse events (AEs) were chosen as the safety endpoint.

### Summary measures and synthesis of results

Review manager 5.4 was used to assess the data. Estimated standard mean differences and estimated risk ratio (standard mean difference [SMD] or risk ratio [RR]; 95% confidence interval [CI]) were calculated using a random effects model. The *I*^*2*^ statistic was used to estimate the statistical heterogeneity as follows: *I*^*2*^ < 30% represents “low heterogeneity,” 30% ≤ *I*^*2*^ ≤ 50% means “moderate heterogeneity” and *I*^*2*^ > 50% means “substantial heterogeneity.” *P*-value < 0.05 was considered to be significant for all analyses, and tests are two-tailed.

### Risk of Bias

The risk-of-bias plot was assessed using Review Manager 5.4 software (The Cochrane Collaboration, Oxford, UK) for individual studies. The unified standard of the Cochrane Collaboration was applied to assess the risk of bias for RCTs that included selection bias, performance bias, detection bias, attrition bias, reporting bias, and other potential biases.

## Results

### Search results

**Fig. 1:**
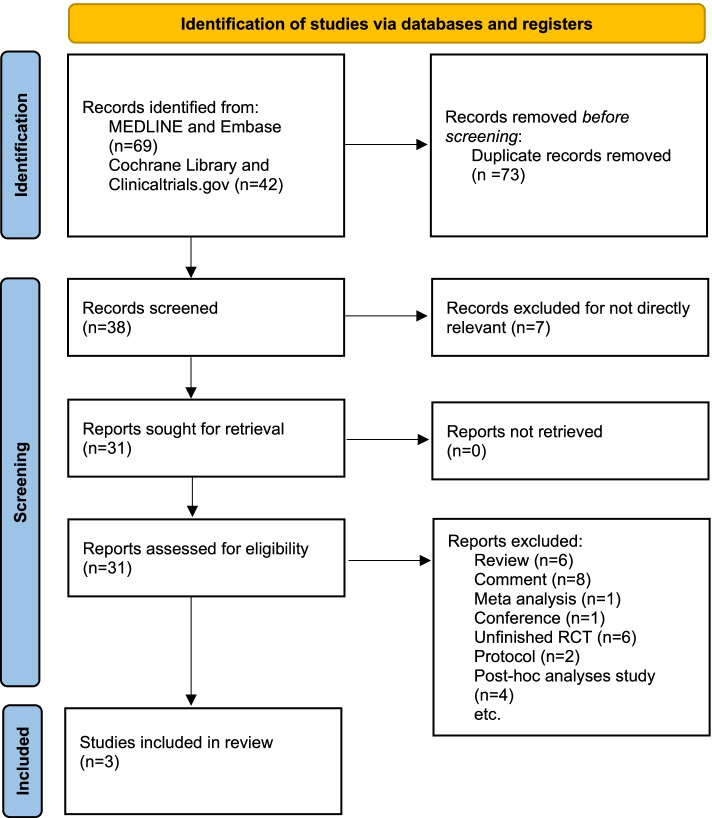
The study search, selection and inclusion process

Sixty-nine studies and abstracts were retrieved from MEDLINE and Embase, as well as 42 studies from the Cochrane Library and clinicaltrials.gov. 73 studies were removed owing to duplication, and 7 studies were eliminated because they were not directly related to the topic, such as studies of other drugs or pathophysiological study of migraine. After removing duplicates and irrelevant articles, 31 articles were directly related to the topic of interest. However, 28 of these articles were excluded because there were 1 conference, 2 protocol, 4 post-hoc analysis studies, 6 unfinished RCTs, 1 meta-analysis (Interventions are not atogepant versus placebo), 8 comments and 6 reviews. Eventually, 3 RCTs were included in our study and related information was shown in supplementary materials S1. The complete search process was detailed in the Fig. 1.

### Primary efficacy outcomes

**Fig. 2:**
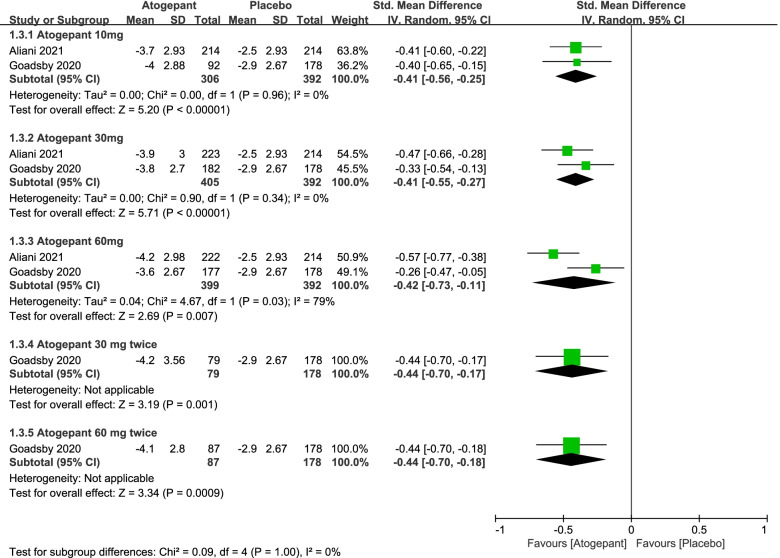
The pooled SMD of monthly migraine days in different doses compared with placebo. The green square indicates the estimated SMD for each RCT. The size of green square indicates the estimated weight of each RCT, and the extending lines indicate the estimated 95% CI of SMD for each RCT. The black diamond indicates the estimated SMD (95% CI) for all patients together. CI, confidence interval; RCT, randomized controlled trial; SMD, standard mean difference

After measurement, we considered one metric in this study as primary outcomes, namely monthly migraine days (Fig. 2). In terms of this, each of the different doses of atogepant group showed a certain amount of advantages. The mean monthly migraine days during 3 months in the atogepant 10 mg group was 0.41 days less than the days in the placebo group (SMD =  − 0.41, 95%CI: [− 0.56, − 0.25], *P* < 0.00001), the atogepant 30 mg group (SMD =  − 0.41, 95%CI: [− 0.55, − 0.27], *P* < 0.00001) and the atogepant 60 mg group (SMD =  − 0.42, 95% CI: [− 0.73, − 0.11], *P* = 0.007) as well.

### Secondary efficacy outcomes

**Fig. 3:**
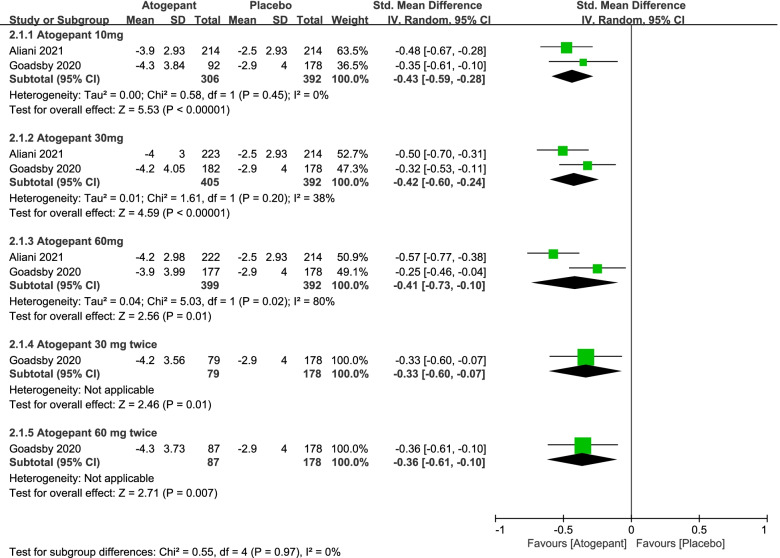
The pooled SMD of monthly headache days in different doses compared with placebo. The green square indicates the estimated SMD for each RCT. The size of green square indicates the estimated weight of each RCT, and the extending lines indicate the estimated 95% CI of SMD for each RCT. The black diamond indicates the estimated SMD (95% CI) for all patients together. CI, confidence interval; RCT, randomized controlled trial; SMD, standard mean difference

**Fig. 4:**
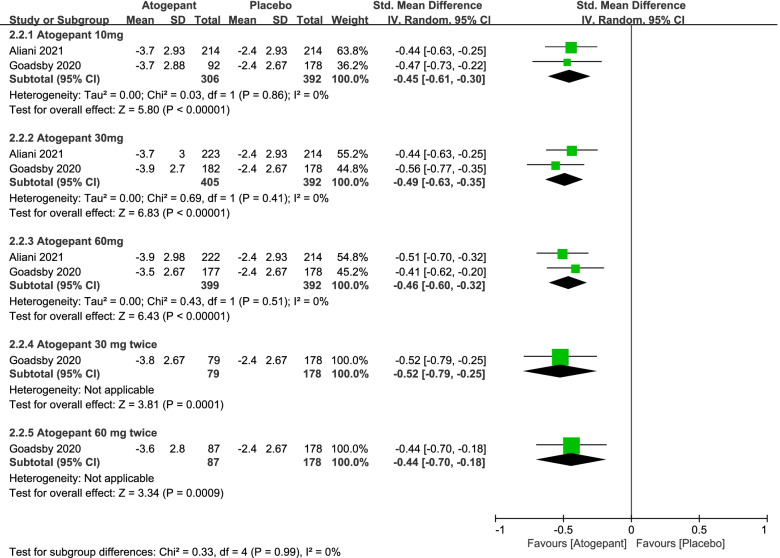
The pooled SMD of monthly medication use days in different doses compared with placebo. The green square indicates the estimated SMD for each RCT. The size of green square indicates the estimated weight of each RCT, and the extending lines indicate the estimated 95% CI of SMD for each RCT. The black diamond indicates the estimated SMD (95% CI) for all patients together. CI, confidence interval; RCT, randomized controlled trial; SMD, standard mean difference

**Fig. 5:**
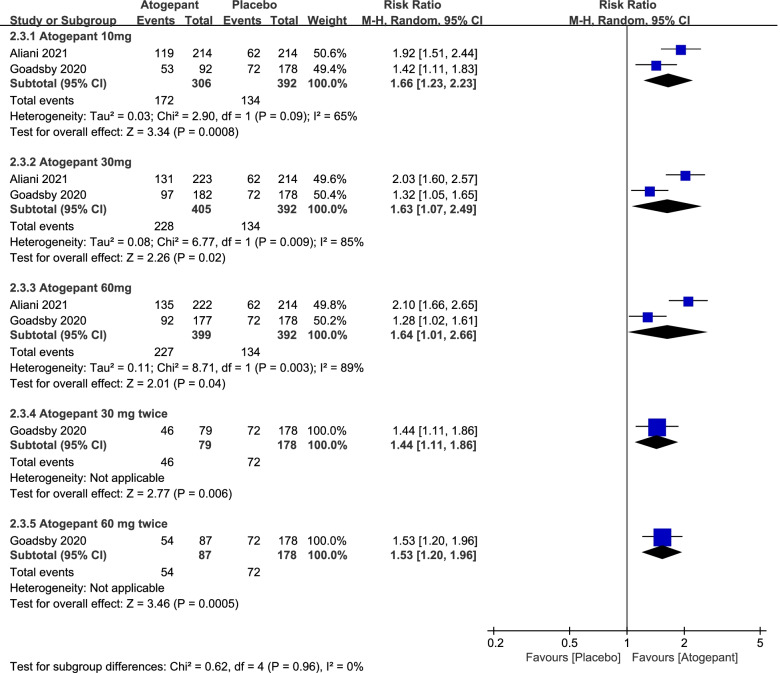
The pooled RR of ≥ 50% reduction in monthly migraine days. The blue square indicates the estimated RR for each RCT. The size of blue square indicates the estimated weight of each RCT, and the extending lines indicate the estimated 95% CI of RR for each RCT. The black diamond indicates the estimated RR (95% CI) for all patients together. CI, confidence interval; RCT, randomized controlled trial; RR: risk ratio

In this part, a number of endpoint measures were also estimated to explore the efficacy of atogepant for migraine, including monthly headache days, acute medication use days per month and ≥ 50% reduction in monthly migraine days (Fig. 3, 4 and 5). It revealed that the mean monthly headache days in the atogepant 10 mg (SMD =  − 0.43, 95%CI: [− 0.59, − 0.28], *P* < 0.00001), 30 mg (SMD =  − 0.42, 95%CI: [− 0.60, − 0.24], *P* < 0.00001), 60 mg once-daily groups (SMD =  − 0.41, 95%CI: [− 0.73, − 0.10], *P* = 0.01) were all less than the days in the placebo group. Moreover, the decrease in the average number of days of medication use monthly in 3 months was also an indication of the change in the number of days of migraine attacks and thus confirmed the preventive effect of the drugs. Specific results were as follows: the atogepant 10 mg group (SMD =  − 0.45, 95%CI: [− 0.61, − 0.30], *P* < 0.00001), the atogepant 30 mg group (SMD =  − 0.49, 95%CI: [− 0.63, − 0.35], *P* < 0.00001), the atogepant 60 mg group (SMD =  − 0.46, 95%CI: [− 0.60, − 0.32], *P* < 0.00001). In regard to the outcomes of ≥ 50% reduction in monthly migraine days, the proportion of patients in the atogepant 10 mg group with a 50% or more reduction in mean migraine days per month during 3 months had a pronounced increase than the placebo group (RR = 1.66, 95%CI: [1.23, 2.23], *P* = 0.0008), after that, the same was true for the atogepant 30 mg group (RR = 1.63, 95%CI: [1.07, 2.49], *P* = 0.02) and the atogepant 60 mg group (RR = 1.64, 95%CI: [1.01, 2.66], *P* = 0.04).

### Safety outcomes

**Fig. 6:**
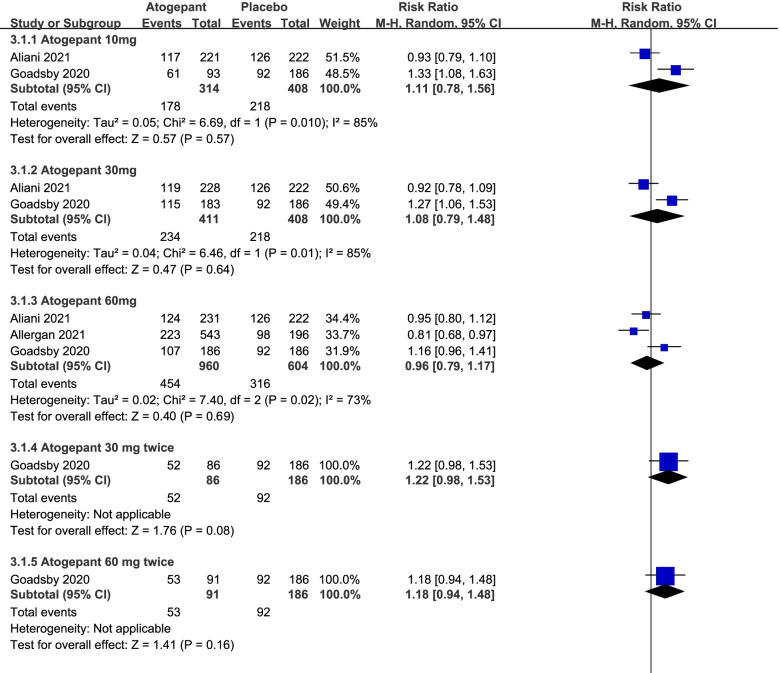
The pooled RR of ≥ 50% reduction in adverse events. The blue square indicates the estimated RR for each RCT. The size of blue square indicates the estimated weight of each RCT, and the extending lines indicate the estimated 95% CI of RR for each RCT. The black diamond indicates the estimated RR (95% CI) for all patients together. CI, confidence interval; RCT, randomized controlled trial; RR: risk ratio

Since the types of adverse events could not be discriminated clearly, only the total number of adverse events was analyzed. As a result, there were no significant differences in outcomes of adverse events between the treatment groups and the placebo group. (atogepant 10 mg: RR = 1.11, 95%CI: [0.78, 1.56], *P* = 0.57, atogepant 30 mg: RR = 1.08, 95%CI: [0.79, 1.48], *P* = 0.64, atogepant 60 mg: RR = 0.96, 95%CI: [0.79, 1.17], *P* = 0.68, Fig. 6).

### Subgroup analysis

We separately conducted a comparative analysis of atogepant 10 mg and 30 mg, 10 mg and 60 mg, 30 mg and 60 mg about their efficacy (monthly migraine days, monthly headache days and monthly medication use days) and safety. The results showed that none of the differences in present outcomes (all *p* > 0.05) (Shown in supplement materials S2 and S3).

### Risk of bias

**Fig. 7:**
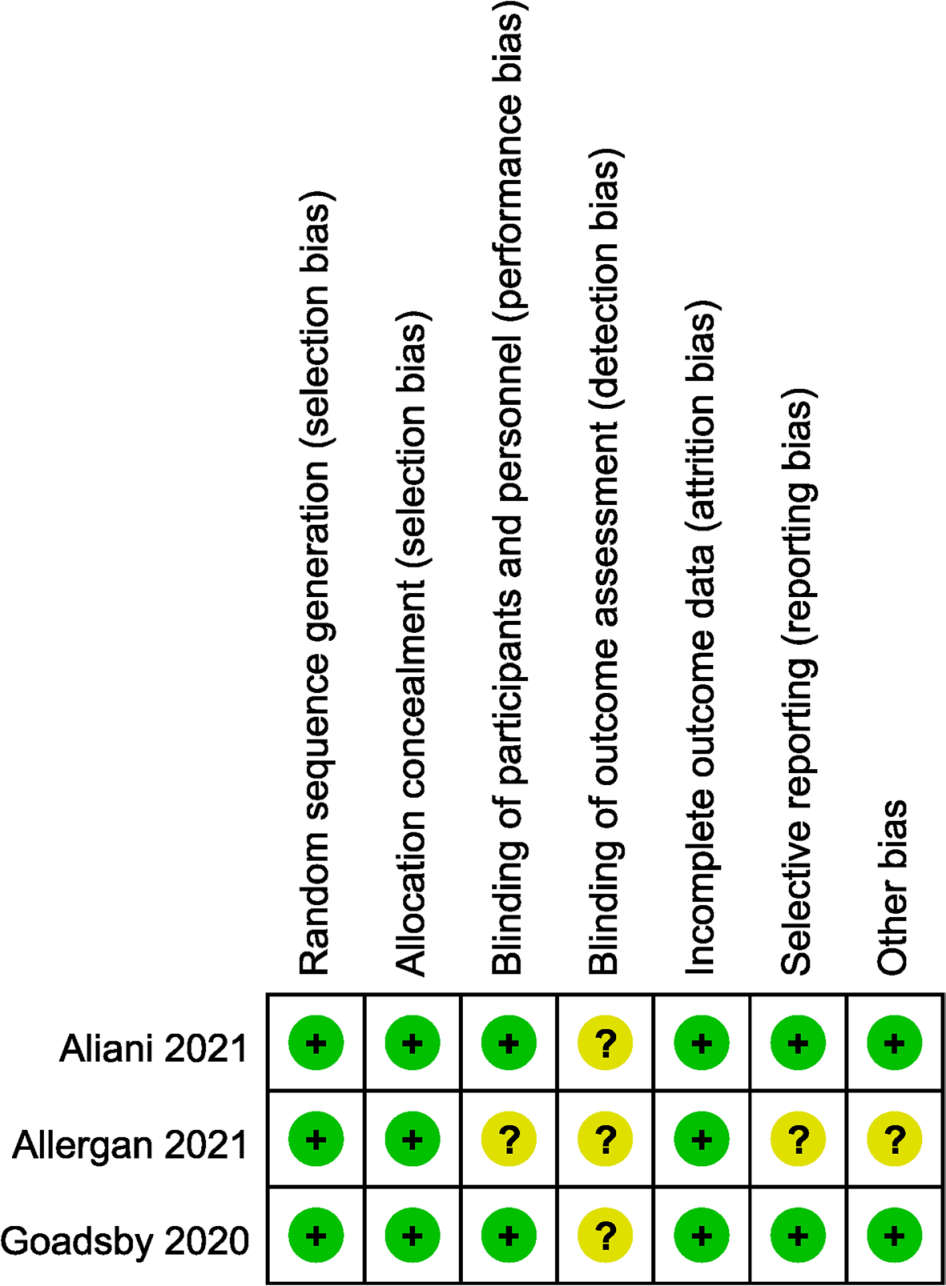
Summary table for potential bias analysis for included study

The independent risk of bias of the three included trials has been appraised with details of Fig. 7. The risks for blinding of outcome assessment (detection bias) are all unclear in the Goadsby et al. 2020 study, Allergan et al. 2021 and Ailani et al. 2021 study. Study conducted by Allergan et al. 2021 had three additional biases that were unclear because the details of the relevant research were not yet available. In addition to the above mentioned, other risks were all low risks of bias in the three trials.

## Discussion

Our study synthetically evaluated the efficacy and safety of atogepant (AGN-241689) which was the newest gepants in nearly 2,466 patients with 4–14 days of migraine per month, including the pivotal Phase 2b/3 study (NCT02848326), the Phase 3 placebo-controlled study (NCT03777059) and the Phase 3 safety study (NCT03700320) [24, 25]. These clinical researches have a very solid academic significance regarding the evaluation of the safety and efficacy of atogepant for the prevention of episodic migraine. On this basis, it is of great significance to perform a systematic evaluation of research findings to improve the level of evidence for evidence-based medicine. Meta-analysis is high-efficiency study designs, which analyzes a study and its results with predefined steps by definition quantitatively [26]. Until our study, no meta-analysis for atogepant based on clinical trials has been conducted and published. Therefore, our study has some groundbreaking significance and may provide some guidance for the subsequent use of atogepant for migraine treatment.

The results of our meta-analysis showed that atogepant is an effective drug for the prevention of EM, compared with placebo. In the primary outcome, mean number of migraine days per month, all tested doses of atogepant were effective in reducing migraine attacks in patients. In controlled studies of preventive treatment, the percentage of participants with at least a 50% reduction in the mean number of migraine days per month over three months was recommended as a surrogate primary endpoint [27]. Therefore, we used the mean number of headache days per month, the number of days of acute migraine attack medication use per month, and the number of half remissions as secondary indicators. Consistent with the results of the underlying study, atogepant did show a significant effect on the prevention of migraine attacks. To our disappointment, significant dose-related changes in efficacy were absent in both the primary and secondary outcomes. In order to further investigate a relatively more appropriate dose, a subgroup analysis was carried out with the comparison between every two groups. Eventually, no significant differences were found. Given that the tolerance and subjective sensation of patients, starting treatment in small doses is probably a choose, in spite of no differences in effectiveness or safety for all doses. Besides, atogepant 30 mg twice daily and 60 mg twice daily also showed corresponding efficacy in the Goadsby et al. 2020 study, but considering that these two doses were not included in the Aliani et al. 2021 study, there may not be a trend to recommend these two doses at this time. Given the integrity of the data, we did not exclude twice-daily doses from this study, but more RCTs are needed to explore their difference in effect from other doses.

With regard to treatment-related AEs, gastrointestinal symptoms such as nausea and constipation are the most common. However, due to the large differences in the types of AEs between the three trials, we were unable to conduct a more detailed analysis. In the overall analysis of all adverse reactions, atogepant showed no significant differences. As an additional note, a recent phase 1 clinical study showed that the combination of multiple daily doses of atogepant 60 mg with a single dose of oral contraceptive ethinyl estradiol 0.03 mg/levonorgestrel 0.15 mg was safe in healthy female, and also had good tolerability [28].

With the exception of gepants, CGRP monoclonal antibodies are equally effective in patients with episodic migraine [29, 30], used to preventive treatment. Atogepant, however, is an orally administered small molecule that has a much shorter half-life and may have the advantage of being more convenient and acceptable for some specific patients. CGRP monoclonal antibodies are large molecules that usually be administered intravenously or by subcutaneous injection. Because of their long half-life (21–48 days), therapeutic concentrations of CGRP monoclonal antibodies can last 3 months or longer [31], which may become a problem in some patients. According to relevant studies, upper respiratory tract infection was the most common adverse event in CGRP monoclonal antibodies [32]. Pain at the injection site was also a non-negligible problem that caused bad somatic sensations in individuals. This is not a concern with oral gepants. Nevertheless, in terms of dosing frequency, the small molecule Atogepant needs to be taken orally every day, which may not be an advantage for some people, compared to monthly or monthly injections [33]. Rimegepant has a similar situation for migraine prevention with 75 mg administered orally every other day as a recommended dose. Consequently, this all needs to be measured in a more integrated way.

Atogepant is a second-generation small molecule CGRP receptor antagonist that has recently received FDA-approval, and there is great scope for research on it. Its performance in comparison with several other preventive drugs deserves to be evaluated from the perspectives of efficacy, safety and economic efficiency. Our study further confirms its efficacy and safety for clinical application, which also brings some benefits to patients with episodic migraine requiring prophylactic treatment.

Inevitably, there were some limitations in our study. The number of RCTs included was small, so there is a lack of comprehensive persuasiveness. And each of the RCTs appeared to have been conducted by the same group of researchers, such that the subjectivity of the researchers caused some bias in the results as well. In addition, the population included in this study was only patients with 4–14 days of headache per month, and the efficacy or safety of chronic migraine patients was not considered. Trials of this type are currently underway and could be a direction for future analysis when more details are refined.

## Conclusion

Given all that, atogepant had shown advantageous efficacy in the treatment of episodic migraine regarding all measures from baseline to end of trials. Its safety had also been validated by comparison with placebo. More studies on atogepant are underway and it is believed that it will show unlimited promise in the future for the treatment of migraine.

## Supplementary Information

**Additional file 1.**

**Additional file 2.**

**Additional file 3.**

## Data Availability

The data that support the findings of this study are available upon request by contacting with the corresponding author. The data are not publicly available due to privacy or ethical restrictions.
