# Supplementary material for: The efficacy and safety of atogepant for the prophylactic treatment of migraine: evidence from randomized controlled trials
Source: J Headache Pain. 2022 Jan 29;23(1):19. doi: 10.1186/s10194-022-01391-2 (PMC8903713; doi:10.1186/s10194-022-01391-2)
Supplement: Supplementary file 1 — Additional file 1. [file 10194_2022_1391_MOESM1_ESM.docx]

| Trials | Goadsby et al 2020  NCT02848326 |
| --- | --- |
| *Inclusion criteria* | 1. Men and women aged 18 to 75 years who had a history of migraine with or without aura, diagnosis for at least a year, and onset before the age of 50 years.  2. Use of acute migraine drugs for 14 days or fewer per 28-day period, including 10 days or fewer of triptan use per 28-day period, in the 3 months before the initial screening visit and during the subsequent 28-day screening period was permitted.  3. Participants were eligible if they self-reported a mean of 4–14 migraine days per month in the 3 months before visit 1, and reported 4–14 migraine days in the 28-day baseline period via an eDiary, on the basis of at least 20 days of data. |
| *Exclusion*  *criteria* | 1. Participants had a mean of 15 or more headache days per month in the 3 months before visit 1 or during the baseline period; had a history of inadequate response to at least three medications prescribed for migraine prevention (including two from different classes)  2. Participants had used opioids or barbiturates more than 2 days per month, triptans or ergots 10 days or more per month, or simple analgesics (eg, aspirin, non-steroidal anti-inflam matory drugs, acetaminophen) 15 days or more per month in the 3 months before visit 1 or during the baseline period.  3. Alanine amino transferase or aspartate amino transferase greater than 1·5 times the upper limit of normal (ULN), total bilirubin greater than 1·5 mg/dL (except for participants with a diagnosis of Gilbert’s disease), serum albumin less than 2·8 g/dL, or estimated glomerular filtration rate less than 30 mL/min per 1·73 m². |
| *Efficacy outcomes* | 1. The primary efficacy measure was change from baseline in mean monthly migraine days across the 12-week treatment period, with baseline defined as the 4-week pretreatment period.  2. Secondary efficacy measures were change from baseline in mean monthly headache days, proportion of participants with at least a 50% reduction in mean monthly migraine days, and change from baseline in mean acute medication use days per month.  3. Analysis of a prespecified tertiary efficacy measure found that a significant treatment effect occurred at the first timepoint measured (4 weeks). |
| *Safety outcomes* | The most common TEAEs were nausea, upper respiratory tract infection, and nasopharyngitis. The overall incidence of treatment-related TEAEs increased with atogepant dose. The most common treatment-related TEAE for all dose groups was nausea. No other treatment-related TEAE appeared to be dose related.  Specific serious TEAEs (one occurrence each) were ureterolithiasis, Hodgkin’s disease, cholecystitis, ureteritis, migraine, elective abortion, major depression, and acetaminophen overdose. None of the serious TEAEs was considered treatment related by investigators. |
| *Conclusions* | The findings from this trial support the preliminary efficacy and tolerability of daily administration of atogepant for the preventive treatment of migraine. The safety and efficacy will continue to be evaluated. |

| Trials | Ailain et al 2021  NCT03777059 |
| --- | --- |
| *Inclusion criteria* | 1. Adults 18 to 80 years of age with 4 to 14 migraine days per month in the 3 months before visit 1 and 4 to 14 migraine days during the 28day baseline period according to an electronic diary were deemed to be appropriate candidates for preventive therapy and were allowed to enroll.  2. Participants had to have at least a 1-year history of migraine with or without aura, diagnosed as specified in the International Classification of Headache Disorders, 3rd edition (ICHD-3), and with migraine onset before 50 years of age. |
| *Exclusion*  *criteria* | 1. Participants had a current diagnosis of chronic migraine, new daily persistent headache, trigeminal autonomic cephalalgia (e.g., cluster headache), or painful cranial neuropathy as defined by the ICHD-3 or if they averaged 15 or more headache days per month across the 3 months before visit 1 or during the 28-day baseline period.  2. Participants had had an inadequate response to more than four oral medications prescribed for the preventive treatment of migraine, two of which needed to have different mechanisms of action.  3. Participants who used opioids or barbiturates on more than 2 days per month, triptans or ergots on 10 or more days per month, or simple analgesic agents (e.g., aspirin, nonsteroidal anti-inflammatory drugs [NSAIDs], or acetaminophen) on 15 or more days per month in the 3 months before visit 1 or during the 28-day baseline period were also excluded.  4. Any female participant who was pregnant, planning to become pregnant, or lactating was excluded. |
| *Efficacy outcomes* | 1. The primary efficacy end point was the change from baseline in the mean number of migraine days per month across the 12-week treatment period (the average of month 1, month 2, and month 3) as recorded in the diaries or reported during visits.  2. Secondary efficacy end points, which were tested in hierarchical order, were the change from baseline in the mean number of headache days per month across the 12-week treatment period; the change from baseline in the mean number of days of use of medication for the treatment of migraine attacks across the 12-week treatment period; a reduction from baseline of at least 50% in the 3-month average of migraine days per month; the change from baseline in the score on the Role Function–Restrictive domain of the MSQ, version 2.1, at week 12; the change from baseline in the mean monthly score on the Performance of Daily Activities domain of the AIM-D across the 12-week treatment period; and the change from baseline in the mean monthly score on the Physical Impairment domain of the AIM-D across the 12-week treatment period. An exploratory analysis of the time course of efficacy for atogepant according to 4-week intervals, which was based on the leastsquares mean change from baseline in the number of migraine days per month over the 12week treatment period, was also conducted. The full list of additional prespecified end points is available in the trial protocol. |
| *Safety outcomes* | Adverse events were reported by the participants throughout the trial and at each 4-week followup visit. Information on adverse events was collected and documented during each clinic visit. Participants could also report adverse events by telephone call between visits. |
| *Conclusions* | In our trial, atogepant once daily was effective for reducing the number of migraine days and headache days in the preventive treatment of migraine over 12 weeks. Adverse events included constipation, nausea, and upper respiratory tract infection. Longer and larger trials are needed to determine the effect and safety of atogepant for migraine prevention. |

| Trials | Allergan et al 2021  NCT03700320 |
| --- | --- |
| *Inclusion criteria* | 1. Written informed consent and participant privacy information (e.g., written authorization for use and release of health and research study information) obtained from the participant prior to initiation of any study-specific procedures.  2. Participant is a candidate to be prescribed at least one of the protocol-defined acceptable oral SOC migraine prevention medications and the participant is willing to accept SOC treatment.  3. Participants must be using a medically acceptable and effective method of birth control during the course of the entire study.  4. At least a 1-year history of migraine with or without aura consistent with a diagnosis.  5. Age of the participant at the time of migraine onset < 50 years.  6. History of 4 to 14 migraine days per month on average in the 3 months prior to Visit 1. |
| *Exclusion*  *criteria* | 1. Difficulty distinguishing migraine headaches from tension-type or other headaches.  2. Has a history of migraine accompanied by diplopia or decreased level of consciousness or retinal migraine.  3. Has a current diagnosis of chronic migraine (CM), new persistent daily headache, trigeminal autonomic cephalgia (e.g., cluster headache), or painful cranial neuropathy.  4. ≥ 15 headache days per month on average across the 3 months prior to Visit 1.  5. Usage of opioids or barbiturates > 2 days/month, triptans or ergots ≥ 10 days/month, or simple analgesics (e.g., aspirin, nonsteroidal anti-inflammatory drugs (NSAIDs), acetaminophen) ≥ 15 days/month in the 3 months prior to Visit 1 per investigator's judgment, or during the baseline period. For all participants, barbiturates are excluded 30 days prior to screening and during the baseline period. For participants randomized to atogepant, barbiturates are excluded through the duration of the study as well.  6. Female participant is pregnant, planning to become pregnant during the course of the study, or currently lactating. Women of childbearing potential must have a negative urine pregnancy test.  7. Any clinically significant hematologic, endocrine, pulmonary, renal, hepatic, gastrointestinal (GI), or neurologic disease.  8. Hypertension as defined by sitting systolic blood pressure (BP) > 160 millimeter of mercury (mm Hg) or sitting diastolic BP > 100 mm Hg at Visits 1 or Visit 2. Vital sign measurements that exceed these limits may be repeated only once.  9. At Visit 1, a user of recreational or illicit drugs or has had a history within the past year of drug or alcohol abuse or dependence.  10. History of any GI prior procedures or GI conditions (e.g., diarrhea syndromes, inflammatory bowel disease) that may affect the absorption or metabolism of atogepant; participants with prior gastric bariatric interventions (e.g., Lap Band) which have been reversed are not excluded. |
| *Safety outcomes* | Primary Outcome Measures: Percentage of Participants With at Least 1 Treatment Emergent Adverse Event (TEAE) [ Time Frame: From first dose up to the end of study (median treatment of 52 weeks) + 4 weeks follow-up ]  An adverse event (AE) is any untoward medical occurrence in a patient or clinical investigation participant administered a pharmaceutical product and which does not necessarily have a causal relationship with this treatment. An AE can therefore be any unintended sign (including an abnormal laboratory finding), symptom, or disease temporally associated with the use of a medicinal (investigational) product, whether or not related to the medicinal (investigational) product. A TEAE is an AE that occurs or worsens after receiving investigational study drug.  Secondary Outcome Measures:  Percentage of Participants With Clinically Significant Laboratory Values as Assessed by the Investigator [ Time Frame: From first dose up to the end of study (median treatment of 52 weeks) + 4 weeks follow-up ]  Laboratory tests included tests of hematology, chemistry, and urinalysis. The investigator determined if the results were potentially clinically significant (PCS). Only categories with at least one participant are reported.  Percentage of Participants With Clinically Significant Electrocardiogram (ECG) Findings as Assessed by the Investigator [ Time Frame: Up to Week 52 ]  A standard 12-lead ECG was performed. The investigator determined if the result was potentially clinically significant. Only categories with at least one participant are reported.  Percentage of Participants With Clinically Significant Vital Sign Measurements as Assessed by the Investigator [ Time Frame: From first dose up to the end of study (median treatment of 52 weeks + 4 weeks follow-up) ]  Vital sign measurements included sitting and standing blood pressure (BP), sitting and standing pulse rate, respiratory rate, temperature, and body weight. The investigator determined if the results were clinically significant. Only categories with at least one participant are reported.  Number of Participants With Most Severe Columbia-Suicide Severity Rating Scale (C-SSRS) Assessing Suicidal Ideation or Suicidal Behavior [ Time Frame: Up to Week 52 ]  The C-SSRS is a clinician-rated instrument that reports the severity of both suicidal ideation and behavior. Suicidal ideation was classified on a 5-item scale: 1 (wish to be dead), 2 (nonspecific active suicidal thoughts), 3 (active suicidal ideation with any methods [not plan] without intent to act), 4 (active suicidal ideation with some intent to act, without specific plan), and 5 (active suicidal ideation with specific plan and intent). Suicidal behavior is classified on a 5-item scale: 0 (no suicidal behavior), 1 (preparatory acts or behavior), 2 (aborted attempt), 3 (interrupted attempt), and 4 (actual attempt). More than 1 classification can be selected provided they represent separate episodes. (Minimum total score 0, maximum total score 5; higher total scores indicate more suicidal ideation and/or suicidal behavior). Only the most severe suicidal ideation and the most severe suicidal behavior counted during the treatment period for at least 1 participant are reported. |
|  |  |
