# Supplementary figures and images for: The efficacy and safety of atogepant for the prophylactic treatment of migraine: evidence from randomized controlled trials
Source: J Headache Pain. 2022 Jan 29;23(1):19. doi: 10.1186/s10194-022-01391-2 (PMC8903713; doi:10.1186/s10194-022-01391-2)

A

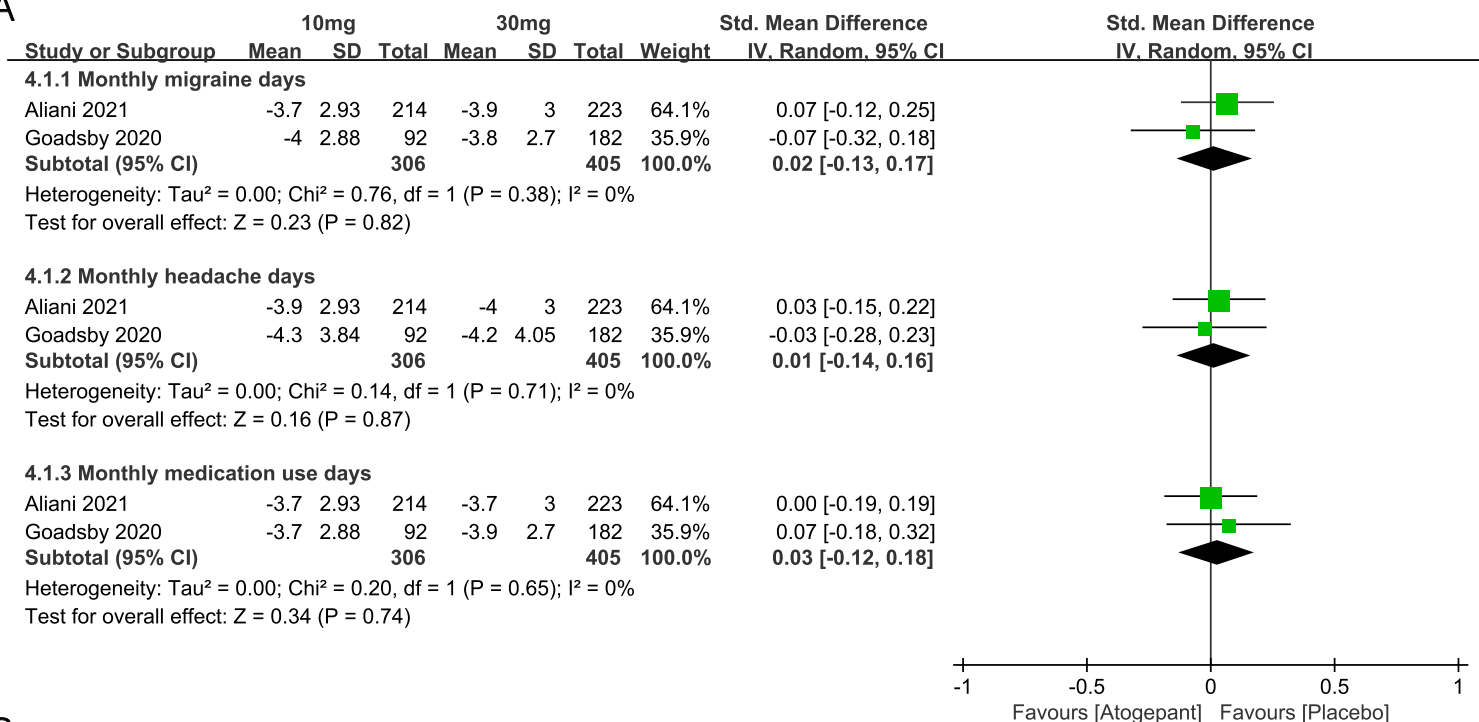

B

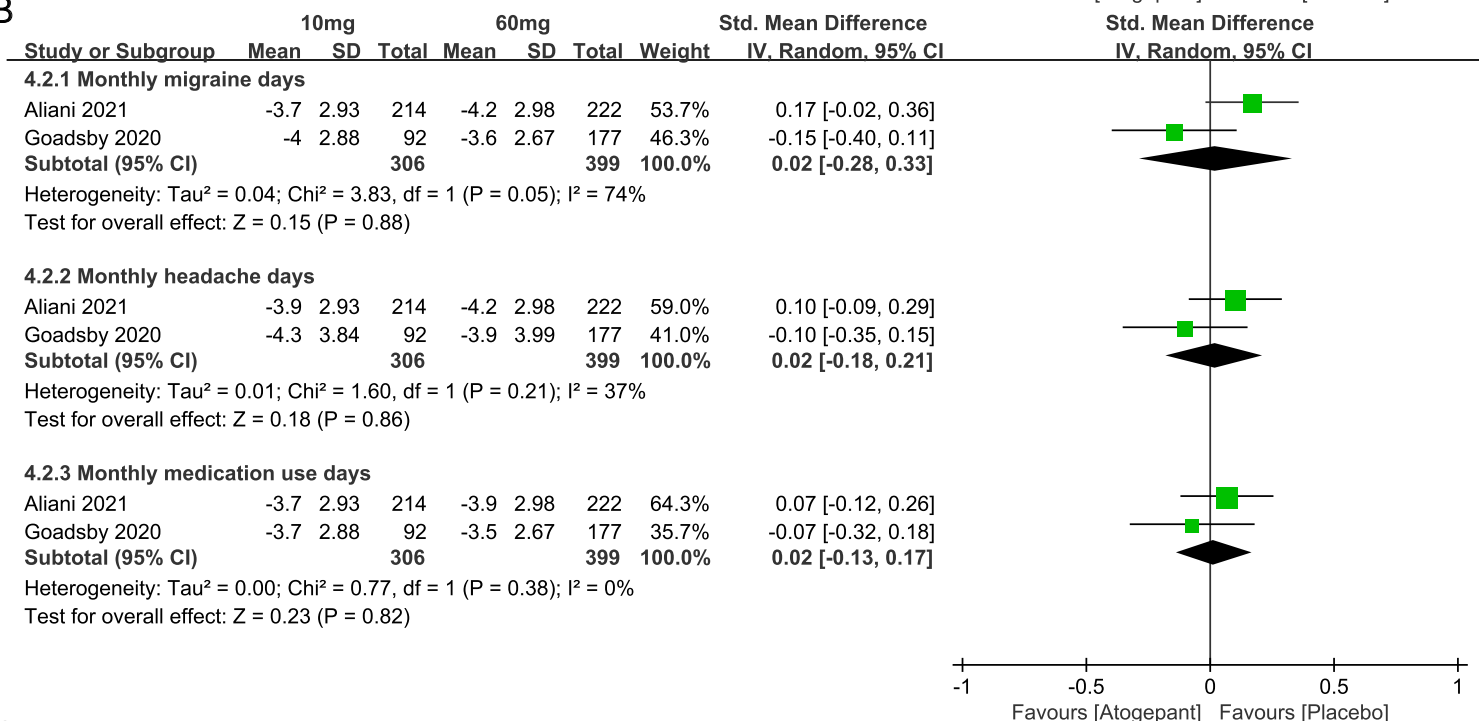

C

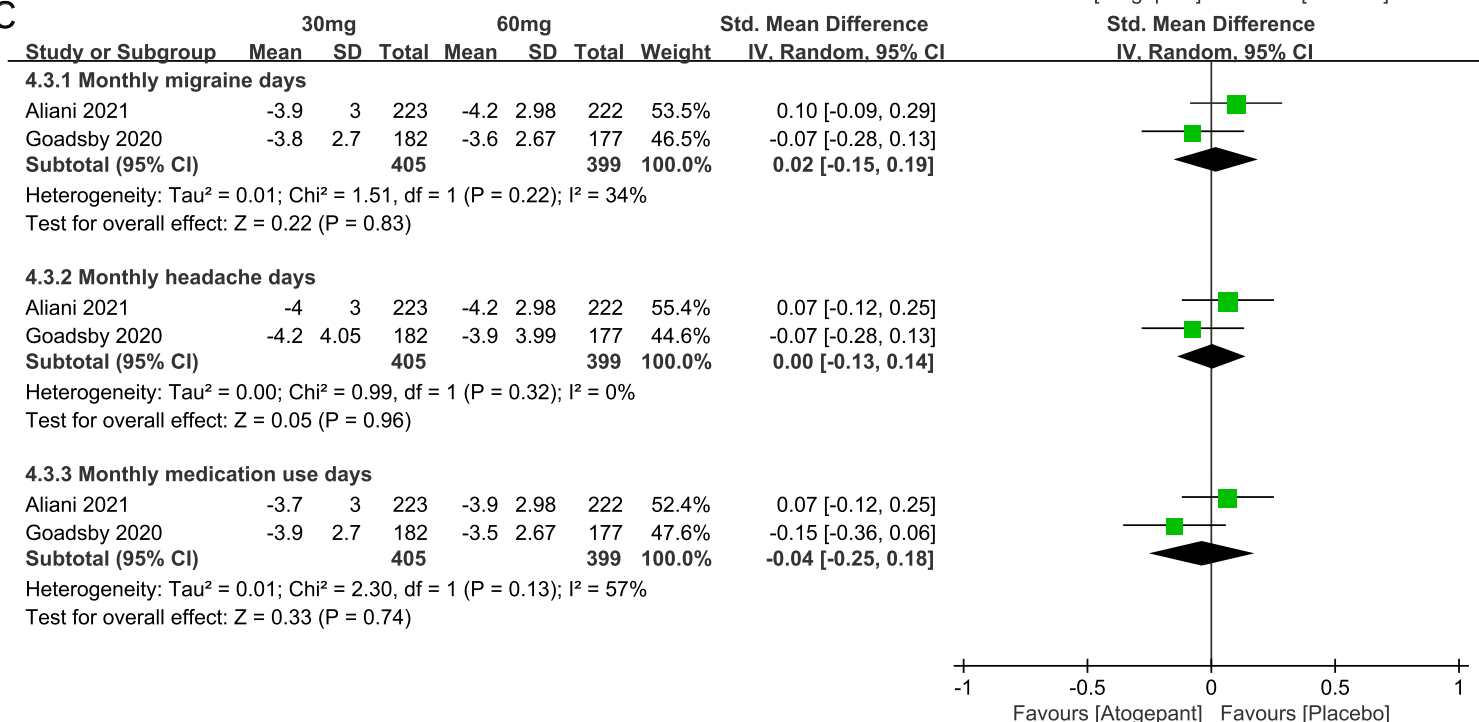

Supplement: Supplementary file 2 — Additional file 2. [file 10194_2022_1391_MOESM2_ESM.pdf]

A

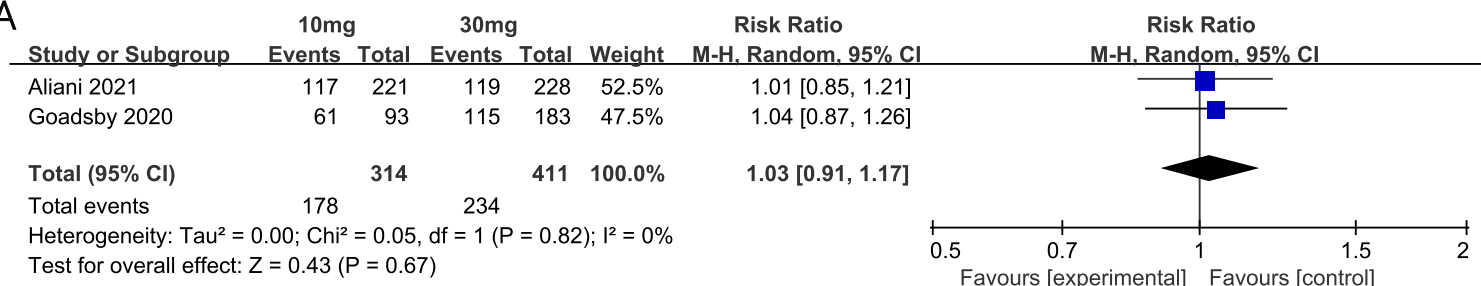

B

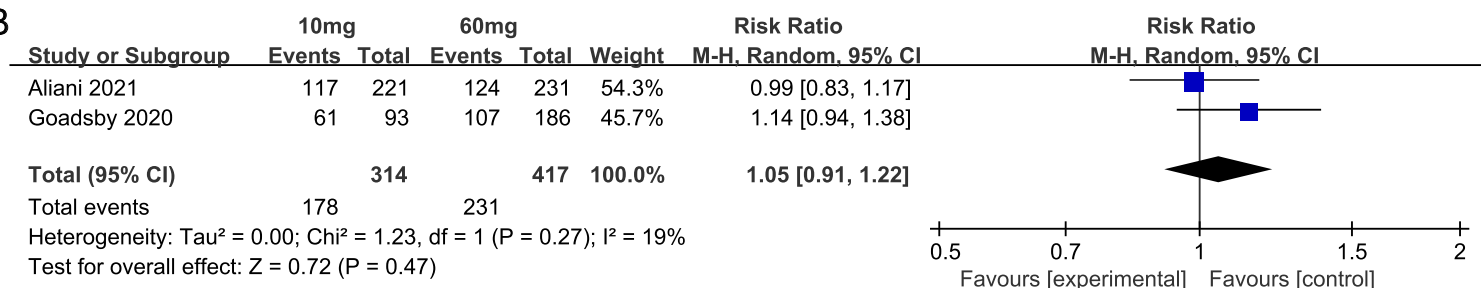

C

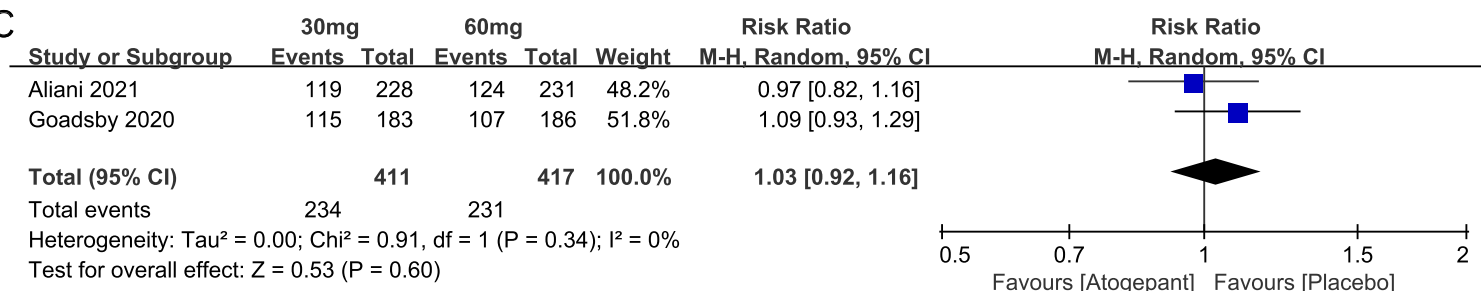

Supplement: Supplementary file 3 — Additional file 3. [file 10194_2022_1391_MOESM3_ESM.pdf]
